# Supplementary material for: Liquid-Plasma Hydrogenated Synthesis of Gray Titania with Engineered Surface Defects and Superior Photocatalytic Activity
Source: Nanomaterials (Basel). 2020 Feb 17;10(2):342. doi: 10.3390/nano10020342 (PMC7075135; doi:10.3390/nano10020342)
Supplement: Supplementary file 1 [file nanomaterials-10-00342-s001.pdf]

Supplementary Materials

Liquid-Plasma Hydrogenated Synthesis of Gray Titania with Engineered Surface Defects and Superior Photocatalytic Activity

Feng Zhang [1,†], Guang Feng [2,3,†], Mengyun Hu [1,3], Yanwei Huang [1,3] and Heping Zeng [1,2,3,4,*]

[1] State Key Laboratory of Precision Spectroscopy, East China Normal University, Shanghai 200062, China; 18843156039@163.com (F.Z.); myhu@phy.ecnu.edu.cn (M.H.); ywhuang@lps.ecnu.edu.cn (Y.H.)
[2] Shanghai Key Laboratory of Modern Optical System, Engineering Research Center of Optical Instrument and System, Ministry of Education, School of Optical-Electrical and Computer Engineering, University of Shanghai for Science and Technology, Shanghai 200093, China; sunnyfeng1992@163.com
[3] Chongqing Institute of East China Normal University, Chongqing 401120, China
[4] CAS Center for Excellence in Ultra-Intense Laser Science, Shanghai 201800, China
* Correspondence: hpzeng@phy.ecnu.edu.cn; Tel.: +86-021-6223-2108
† These authors contributed equally to this work.

Table S1. The integrated peak area of the diffraction peak at (101) facet.

Samples	Power	Time for Electrolysis	Area	FWHM
TiO_2	0W	0h	232996	0.448
Ti360	360W	1h	225421	0.449
Ti420	420 W	1h	232567	0.449
Ti480	480 W	1h	228942	0.449

The integrated peak area and FWHM of peak at (101) facet for all samples were calculated as shown in Table S1, and there is nearly no differences in both integrated peak and FWHM data among these samples.

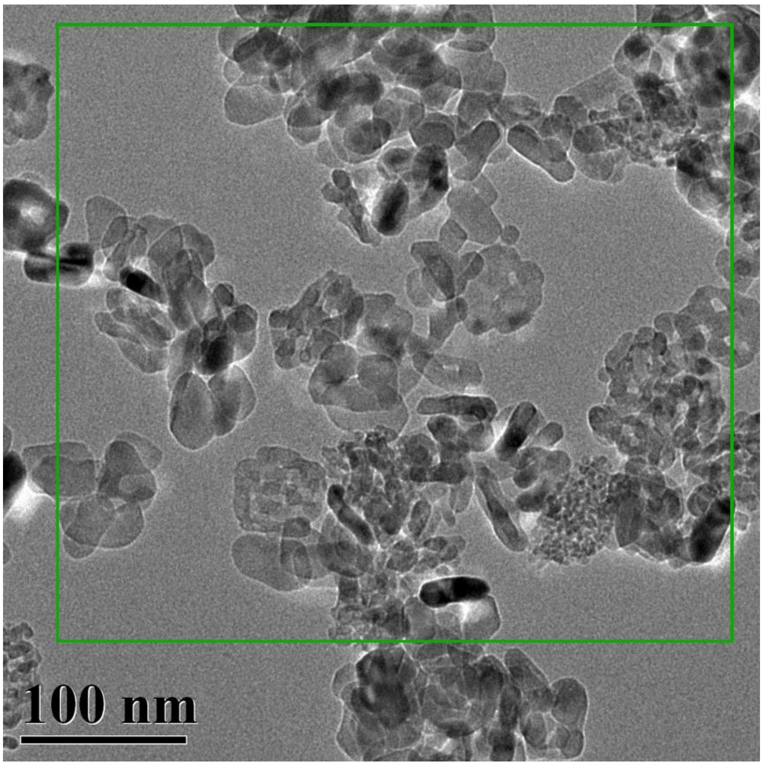

(A)

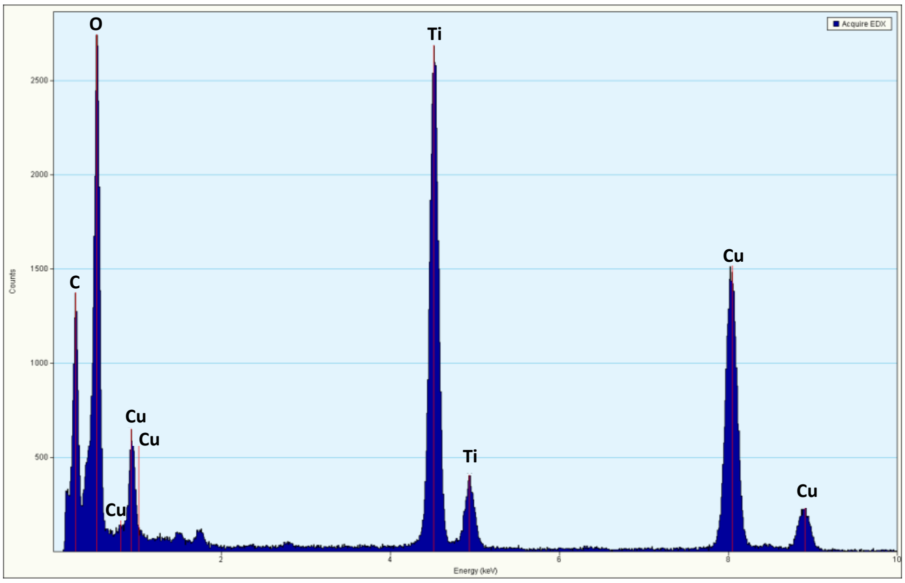

(B)

Figure S1. (A) The selected area for elements analysis in TEM of Ti420, and (B) the EDS results.

The EDS results of Ti420 confirm the existences of the elements of Ti, O, Cu, and C. Elements of C and Cu belong to carbon support film, which implies no other impurities in gray TiO_{2-x}.
